# Association of arterial stiffness with single nucleotide polymorphism rs1333049 and metabolic risk factors

**DOI:** 10.1186/1475-2840-12-93

**Published:** 2013-06-21

**Authors:** Suphawadee Phababpha, Upa Kukongviriyapan, Poungrat Pakdeechote, Laddawan Senggunprai, Veerapol Kukongviriyapan, Chatri Settasatian, Pyatat Tatsanavivat, Phongsak Intharaphet, Vichai Senthong, Nantarat Komanasin, Nongnuch Settasatian, Stephen E Greenwald

**Affiliations:** 1 Department of Physiology, Faculty of Medicine, Khon Kaen University, Khon Kaen, Thailand; 2 Department of Pharmacology, Faculty of Medicine, Khon Kaen University, Khon Kaen, Thailand; 3 Department of Pathology, Faculty of Medicine, Khon Kaen University, Khon Kaen, Thailand; 4 Department of Medicine and Queen Sirikit Heart Center of the Northeast, Faculty of Medicine, Khon Kaen University, Khon Kaen, Thailand; 5 Faculty of Associated Medical Sciences, Khon Kaen University, Khon Kaen, Thailand; 6 Blizard Institute, Barts & The London School of Medicine & Dentistry, Queen Mary University of London, London, United Kingdom

**Keywords:** Metabolic syndrome, Pulse wave velocity, Arterial stiffness, Chromosome 9p21.3, Single nucleotide polymorphism rs1333049

## Abstract

**Background:**

Increased arterial stiffness is a cardiovascular outcome of metabolic syndrome (MetS). The chromosome 9p21 locus has been identified as a major locus for risk of coronary artery disease (CAD). The single nucleotide polymorphism (SNP), rs1333049 on chromosome 9p21.3 has been strongly associated with CAD and myocardial infarction. Increased arterial stiffness could be the link between the 9p21 polymorphism and increased cardiovascular risk. Since the impact of a genetic polymorphism on arterial stiffness especially in Asian populations has not been well defined, we aimed to investigate the association of arterial stiffness with rs 1333049 variant on chromosome 9p21.3 in Thai subjects with and without MetS risk factors.

**Methods:**

A total of 208 Thai subjects, aged 35–75 years, 135 with and 73 without MetS, according to IDF and NCEP-ATPIII criteria, were included in this study. Aortic-femoral pulse wave velocity (afPWV), brachial-ankle pulse wave velocity (baPWV) and aortic ankle pulse wave velocity (aaPWV) were measured and used as markers of arterial stiffness. The chromosome 9p21.3 locus, represented by the rs 1333049 variant and blood biochemistry were evaluated.

**Results:**

Arterial stiffness was elevated in subjects with MetS when compared with nonMetS subjects. PWV, especially afPWV increased progressively with increasing number of MetS risk factors (r = 0.322, P <0.001). We also found that the frequency distribution of the rs1333049 genotypes is significantly associated with the afPWV (P <0.05). In multivariate analyses, there was an association between homozygous C allele and afPWV (Odds ratio (OR), 8.16; 95% confidence interval (CI), 1.91 to 34.90; P = 0.005), while the GC genotype was not related to afPWV (OR, 1.79; 95% CI, 0.84 to 3.77; P = 0.129) when compared with the GG genotype.

**Conclusions:**

Our findings demonstrate for the first time that arterial stiffness is associated with genetic polymorphism in 9p21 and metabolic risk factors in a Thai population.

## Background

Metabolic syndrome (MetS) is defined as a clustering of metabolic abnormalities including central obesity, hyperglycaemia, dyslipidaemia, hypertriglyceridaemia, and elevated blood pressure [1]. MetS is now a major public health problem with high prevalence in both developed and developing countries [2]. Current estimates suggest that, worldwide, about 20-30% of the population have some form of this syndrome [3]. In Thailand, by 2006, the prevalence of the MetS was about 19% in men and 27% in women [4]. Although environmental risk factors contribute greatly to the development of MetS, genetic factors also play an important role in its pathogenesis [5-8]. A previous study in a Caribbean-Hispanic population found that genetic factors contribute 24% to the MetS after adjusting for age and sex [9]. In the past year, several genome wide association studies have reported associations between a region on chromosome 9p21.3 and risk of cardiovascular disease (CVD) [5]. The same chromosomal region is also associated with type 2 diabetes (T2D) [10]. MetS is strongly associated with the risk of CVD and T2D [11]. People with MetS have higher all cause and cardiovascular mortality than those without MetS.

Increased arterial stiffness, a pathological condition associated with vascular damage and or remodelling, is a cardiovascular outcome of MetS [12-14]. It has been shown to predict CVD morbidity and mortality in the general population and also in T2D [12]. The SNP rs1333049 on chromosome 9p21.3 has a particularly strong association with carotid plaque formation [15], abdominal aortic aneurysm [16], and predicts severity of CAD [17]. Although the effect of MetS and its components on arterial stiffness has been reported in the past, this association has not been addressed in a Thai population. Moreover, there has been little work describing the association of genetic variation on chromosome 9p21.3 with arterial stiffness, especially in Asian nationals [18]. To shed light on a possible influence of these genetic polymorphisms on arterial wall integrity, it is therefore the aim of this study to investigate the association of rs 1333049 variant on chromosome 9p21 and arterial stiffness in Thais with and without MetS risk factors.

## Methods

### Study population

A total of 208 subjects, 117 men and 91 women, aged 35–75 years, were recruited from the Outpatient Clinic of Queen Sirikit Heart Center of Northeast Thailand, Faculty of Medicine, Khon Kaen University, Khon Kaen Province, Thailand. MetS was defined using a combination of the International Diabetes Federation (IDF) and National Cholesterol Education Program Adult Treatment Panel III (NCEP-ATPIII) criteria, with modification of waist circumference (WC) for Asians [3,19,20]. This definition requires at least three of the following components: (1) central obesity (WC ≥ 90 cm in men, and ≥ 80 cm in women), (2) triglyceride concentration ≥ 150 mg/dL or on triglyceride-lowering medication, (3) high-density lipoprotein cholesterol (HDL-c) < 40 mg/dL in men and < 50 mg/dL in women or on medication for low HDL-cholesterol, (4) systolic blood pressure ≥ 130 mmHg and/or diastolic blood pressure ≥ 85 mmHg or on antihypertensive medication, and (5) fasting glucose concentration ≥ 100 mg/dL or drug treatment for elevated glucose. NonMetS (healthy) individuals were defined as those without history or diagnosis of MetS.

Informed consent was obtained from each participant. The study protocol was approved by the Ethics Committee for Human Research of Khon Kaen University (HE510414) and the study was conducted in accordance with the Declaration of Helsinki.

### Blood sample collection and anthropometric parameters

After 12-hour overnight fasting, blood was drawn from an antecubital vein in the morning, and sent for analysis within 3 hours. Biochemical markers in serum such as fasting glucose, lipid profiles, and uric acid were analysed by a Hitachi 917 automatic analyser (Roche Diagnostics, Basel, Switzerland) at the Clinical Laboratory Unit of Queen Sirikit Heart Center of Northeastern Thailand. High sensitivity C-reactive protein (hsCRP) concentration was determined by means of high sensitivity particle enhanced immunonephelometry using the BN ProSpec® System (Siemens Healthcare Diagnostics Products GmbH, Marburg, Germany). Fasting serum insulin was determined by a sandwich immunoassay with commercial kits (Cisbio Bioassays, MA, USA). Insulin resistance was estimated using the homeostasis model assessment of insulin resistance (HOMA-IR) index, calculated from the following expression: HOMA-IR = fasting insulin (μU/mL) x fasting plasma glucose (mmol/L)/22.5. Body mass index (BMI) was calculated as weight (kg) divided by height (m)^2^. WC was measured midway between the inferior margin of the last rib and the iliac crest. Blood pressure was measured in the left arm of supine subjects after 15 minutes of rest, using a Dinamap Carescape v100 (GE Medical System, WI, USA).

### Pulse wave velocity measurement

After blood pressure measurement, PWV was measured according to a previously described method [21] using a custom-built data acquisition system (Arterial Compliance Monitor, Barts and The London’s School of Medicine and Dentistry, UK), compatible with commercial continuous wave Doppler probes (Dopplex MDII, Huntleigh Healthcare, Cardiff, UK) and custom-made reflectance photoplethysmography (PPG) probes. PWV was measured by placing two pulse sensing probes at either end of arterial segment under study. Aortic-femoral pulse wave velocity (afPWV), a good marker of aortic stiffness , was measured by placing the proximal Doppler probe (4 MHz) in the supraclavicular fossa of the subject’s neck at 45° to the subclavian arterial wall, angled so that the ultrasound beam was directed toward the aortic arch. The distal probe (reflectance PPG) was placed over the femoral artery near the inguinal ligament. For brachial-ankle pulse wave velocity (baPWV), the two PPG probes were placed over the brachial artery and dorsalis pedis artery, respectively. For the aortic-ankle pulse wave velocity (aaPWV), the proximal Doppler probe (4-MHz) was placed in the supraclavicular fossa and the distal PPG probe was placed on the foot over the dorsalis pedis artery. The pulse-wave contours from the proximal and distal probes were captured at a sample rate of 1 kHz and recorded for at least 3 min. Data were analysed offline by custom-written software to detect the feet of the proximal and distal pulse waves and to measure the time delay between them. Knowing the distance between the proximal and distal sites and the propagation time delay between the artery waveforms, PWV was calculated and expressed as m/s. We have previously found [21] that the coefficients of variation values of PWV between the intra- and inter-day measurements were less than 5%.

### DNA preparation and genotyping

The genotyping was performed in 171 subjects and these were taken as a representative sample of the entire study population. DNA was extracted from EDTA treated blood samples using a Genomic DNA mini kit (Geneaid, Taiwan) according to the manufacturer’s protocol. Genotyping for rs1333049 was performed using the TaqMan SNP allelic discrimination genotyping assay (Applied Biosystems, CA, USA). A total of 20 ng purified DNA was amplified in a final volume of 20 μL per well in 96 well plates. The PCR conditions were; initial denaturation at 95°C for 10 min, followed by 40 cycles at 92°C for 15 sec and 60°C for 1 min. Post PCR allelic discrimination was carried out by measuring allele-specific fluorescence on an ABI prism® 7500 Sequence Detection System (Applied Biosystems) using the Sequence Detection System software version 1.1. Genotyping was successful in 96% of rs1333049 polymorphism.

### Statistical analyses

Statistical comparison between MetS and nonMetS groups was assessed by Student’s *t*-test or the Mann–Whitney U test as appropriate. Pearson correlation analysis was used to assess relationships between PWV and anthropometric variables, and also between PWV and blood biochemistry. Comparisons among three or more groups were evaluated by one way analysis of variance (ANOVA) followed by Newman-Keuls post-hoc analysis. Multiple logistic regression analysis was employed to identify the risk factors associated with afPWV. Statistical analyses were performed with Stata version 10 (Stata Corp., College Station, TX, USA). A value of *P* <0.05 was considered as significant.

## Results

### Characteristics of the study population

The baseline characteristics of subjects are shown in Table 1. Their mean age was of 59.5 ± 10.3 years (range 35 to 75), 117 (56%) participants were male. Among them, 135 participants were classified as MetS and 73, as nonMetS. According to the IDF and NCEP-ATPIII criteria, WC, systolic blood pressure, triglyceride, fasting blood glucose, insulin, HOMA-IR were higher whereas HDL-cholesterol was lower in MetS subjects. Moreover, age, BMI, pulse pressure, and some parameters of blood biochemistry, including hsCRP and uric acid were significantly higher in MetS than nonMetS subjects.

**Table 1:** Characteristics of the study subjects

**Parameters**	**All subjects(*n *= 208)**	**NonMetS(*n *= 73)**	**MetS(*n *= 135)**	***P*value**
Age (y)	59.5 ± 10.3	55.3 ± 11.3	61.7 ± 9.0	<0.001
Gender (male (%))	117 (56)	47 (64)	70 (52)	0.136
Waist circumference (cm)	88.6 ± 10.4	81.3 ± 9.5	92.5 ± 8.5	<0.001
Body mass index (kg/m^2^)	25.0 ± 3.7	23.1 ± 3.2	26.1 ± 3.4	<0.001
Systolic blood pressure (mmHg)	129 ± 17.0	121 ± 11.2	134 ± 17.7	<0.001
Diastolic blood pressure (mmHg)	73.6 ± 11.1	71.8 ± 9.6	74.6 ± 11.8	0.084
Pulse pressure (mmHg)	55.7 ± 13.7	48.7 ± 9.3	59.5 ± 14.2	<0.001
Heart rate (beats/min)	66.4 ± 12.0	66.4 ± 13.8	66.4 ± 11.0	0.880
Total cholesterol (mg/dL)	184 ± 45.9	180 ± 45.7	186 ± 46.0	0.396
Triglyceride (mg/dL)	181 ± 104	132 ± 66.0	208 ± 112	<0.001
HDL-cholesterol (mg/dL)	43.7 ± 12.6	48.7 ± 13.3	41.0 ± 11.4	<0.001
LDL-cholesterol (mg/dL)	104 ± 38.6	105 ± 40.9	104 ± 37.4	0.805
Fasting glucose (mg/dL)	108 ± 45.9	89.9 ± 17.0	119 ± 53.2	<0.001
Insulin (μIU/mL)	16.2 ± 9.6	13.3 ± 7.8	17.8 ± 10.1	<0.001
HOMA-IR	4.65 ± 4.6	2.96 ± 1.9	5.58 ± 5.3	<0.001
hsCRP (mg/L)	1.79 ± 2.0	1.44 ± 1.6	1.98 ± 2.1	0.015
Uric acid (mg/dL)	6.32 ± 1.8	5.90 ± 2.0	6.54 ± 1.7	0.003

All data are expressed as mean ± SD or number (%). *HDL*, high-density lipoprotein; *LDL*, low-density lipoprotein; *HOMA-IR*, homeostatic model assessment-insulin resistance; *hsCRP*, high sensitivity C reactive protein. *P* value, nonMetS *vs*. MetS.

### PWV and relationship between anthropometry and blood biochemistry

**Figure 1:**
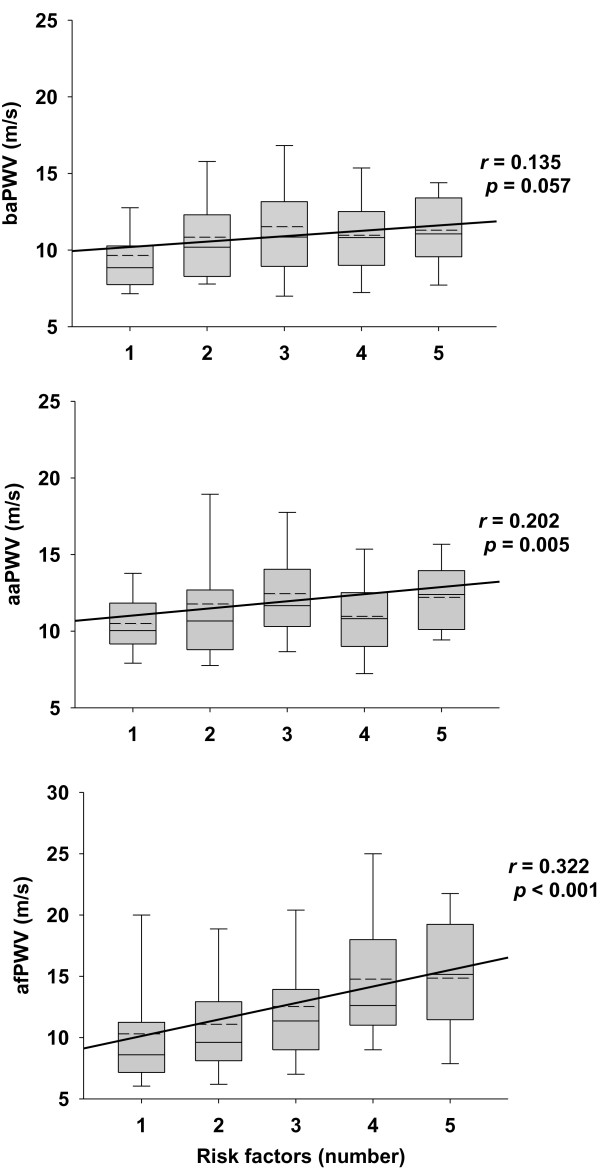
**Relationship between the number of metabolic risk factors and pulse wave velocity for each arterial segment.** The solid line in each box represents the median value, short dashed line represents the mean, the top and bottom of the box represent the interquartile range, and the whiskers indicate the 5th and 95th percentiles. The regression line was derived from the relationship between the number of metabolic risk factors and PWV.

PWV values of each arterial segment (i.e. baPWV, aaPWV, and afPWV) were significantly higher in MetS than nonMetS subjects (Table 2). PWV at all three sites was significantly correlated with age, systolic blood pressure, and WC (Table 3). However, afPWV was more strongly correlated with variables such as BMI, fasting glucose, HOMA-IR, hsCRP and uric acid (Table 3) than the PWV values measured in the arm and the leg. To discern the relationship between PWV and MetS, the relationship between PWV and the number of metabolic risk factors for MetS was analysed, i.e. the presence of insulin resistance, high blood pressure, high triglyceride, low HDL-c, and high WC. baPWV, aaPWV and afPWV increased with increasing number of MetS risk factors (Figure 1). afPWV and aaPWV were significantly correlated with the number of MetS risk factors, the strongest correlation being with afPWV.

**Table 2:** Pulse wave velocities of each arterial segment in non-metabolic and metabolic syndrome subjects

**Pulse wave velocity**	**NonMetS (n=73)**	**MetS (n=135)**	***P*value**
baPWV (m/s)	10.22 ± 3.2	11.25 ± 3.3	0.004
aaPWV (m/s)	11.08 ± 3.2	12.40 ± 2.8	<0.001
afPWV (m/s)	10.64 ± 4.9	13.88 ± 5.4	<0.001

All data are expressed as mean ± SD, baPWV, brachial-ankle pulse wave velocity; aaPWV, aortic-ankle pulse wave velocity; afPWV, aortic-femoral pulse wave velocity. *P* value, nonMetS *vs*. MetS.

**Table 3:** Correlations between pulse wave velocities and anthropometric and blood chemistry variables

**Parameters**	**baPWV**	**aaPWV**	**afPWV**
Age (years)	0.291†	0.358†	0.277†
Systolic blood pressure (mmHg)	0.212*	0.332†	0.242†
Diastolic blood pressure (mmHg)	0.153	0.140	0.139
Waist circumference (cm)	0.162*	0.197*	0.352†
Body mass index (kg/m^2^)	−0.011	0.015	0.257†
Triglyceride (mg/dL)	0.001	−0.027	0.129
HDL-cholesterol (mg/dL)	−0.065	−0.074	−0.114
Fasting glucose (mg/dL)	−0.041	0.060	0.172*
HOMA-IR	−0.012	0.108	0.166*
hsCRP (mg/L)	0.060	0.116	0.225*
Uric acid (mg/dL)	0.061	0.150	0.203*

HDL, high-density lipoprotein; *HOMA-IR*, homeostatic model assessment-insulin resistance; *hsCRP*, high sensitivity C-reactive protein; *baPWV*, brachial-ankle pulse wave velocity; aaPWV, aortic-ankle pulse wave velocity; *afPWV*, aortic-femoral pulse wave velocity. * *P* <0.05; † *P* <0.001.

### Polymorphism of rs1333049 in relation to PWV

The polymorphism of rs1333049 G>C has been reported to be associated with increased risk of CVD. We wished to determine whether the SNP could affect arterial elasticity and thus subsequently increase the likelihood of symptomatic CVD. The SNP rs1333049 G>C was analysed in 171 subjects (114 MetS and 57 nonMetS). The frequencies of the rs1333049 “G” and “C” alleles in all subjects were 66.5% and 33.5%, respectively. In MetS subjects, the frequencies of the “G” and “C” alleles were 68.5% and 31.4%, respectively and were not significantly different from nonMetS subjects. In all subjects taken together, the frequency of the genotypes GG, GC and CC were 46%, 41% and 13%, respectively, the distribution being consistent with the Hardy-Weinberg equilibrium, with Chi-squared = 0.769, P = 0.681.

The influence of rs1333049 polymorphism on arterial elasticity was analyzed. There was an association of rs1333049 genotypes with afPWV, but not with baPWV or aaPWV. The effect of this was further analysed by assigning subjects to a ‘low’ group if their PWV was less than or equal to 11.0 m/s (based on the mean afPWV of nonMetS subjects) and to a ‘high’ group, if greater than 11.0 m/s. The genotype of rs1333049 distribution in the low and high PWV subjects is shown in the Table 4. The distribution was significantly different between the low and high afPWV groups with Chi-squared = 7.45, P <0.05, suggesting a positive association between the rs1333049 SNP and increased arterial stiffness.

**Table 4:** Distribution of rs1333049 genotypes in relation to afPWV

**Aortic**-**femoral** **pulse wave velocity**	**Number subjects (%)**			**Total subjects**
	**GG**	**GC**	**CC**	
< 11 m/s	40 (57)	27 (39)	3 (4)	70
≥11 m/s	39 (39)	45 (45)	17 (17)	101

Chi-square tests of goodness of fit, Chi-square with continuity correction = 7.45, *P* <0.05.

### Multivariate analysis of factors affecting PWV

Since the effect of age, gender, risk factors and rs1333049 genotypes on PWV was strongest in the aorto-femoral segment, we subjected these data to a more detailed analysis. In univariate tests, afPWV was significantly associated with age, risk factors and genotypes (Table 5). Similarly, in multivariate analysis, increased afPWV was associated with the advance in age and increment in the number of MetS risk factors as shown, by the increased OR to 11.12 (95% CI: 2.20-56.18) when risk factors comprised 5 components. Interestingly, afPWV was also increased in relation to the gene dose (“C”) effect (Table 5). The presence of the “GC” or “CC” genotype was associated with increased risk of developing high PWV with OR of 1.79 (95% CI: 0.84-3.77) and 8.16 (95% CI: 19.1-34.90) after adjusting for gender, age and metabolic risk factors.

**Table 5:** Univariate and multivariate logistic regression models for predicting the central arterial stiffness in the study subjects

**afPWV**	**Univariate model**			**Multivariate model**		
		**Odds ratio**	**95% CI**	***P***	**Odds ratio**	**95% CI**	***P***
Gender Female: Male	0.812	0.44 - 1.49	0.50	0.75	0.35-1.58	0.50
Age						
Quartile 1 (≤ 51 years)	1			1		
Quartile 2 (52–59 years)	4.06	1.71 - 9.61	0.001	2.27	0.81 – 6.36	0.120
Quartile 3 (60–66 years)	4.71	1.85 - 11.99	0.001	2.55	0.78 – 8.40	0.123
Quartile 4 (≥ 67 years)	11.69	4.24 - 32.24	<0.001	7.88	2.45 – 25.3	0.001
Risk factor						
≤ 1	1			1		
2	1.83	0.63 - 5.34	0.267	1.36	0.35 - 5.23	0.685
3	3.38	1.33 - 8.59	0.011	2.42	0.74 - 7.91	0.144
4	7.71	2.89 - 20.55	<0.001	4.49	1.33 - 15.14	0.015
5	10.00	2.68 - 37.38	0.001	11.12	2.20 - 56.18	0.004
rs1333049						
GG genotype	1			1		
GC genotype	1.71	0.89 - 3.27	0.106	1.79	0.84 - 3.77	0.129
CC genotype	5.81	1.58 - 21.42	0.008	8.16	1.91 - 34.90	0.005

afPWV, aortic-femoral pulse wave velocity.

## Discussion

This study demonstrates, for the first time, that SNP rs1333049 on chromosome 9p21.3 is an important determinant for the progression of increasing arterial stiffness, especially that of the aorta, in a Thai population. Increased afPWV and aaPWV are associated with increasing number of MetS risk factors, indicating that a clustering of MetS risk may interact synergistically to affect central arterial stiffness. Previous studies have suggested that arterial stiffening in MetS, particularly in diabetic individuals, is associated with elastic lamellae fragmentation and calcification, increased collagen cross-linking, low-grade inflammation, increased smooth muscle basal tone and endothelial dysfunction [22-25]. These processes may be influenced by both genetic and environmental factors [6-8,23]. As the integrity of the aortic wall is dependent on effective load bearing by the elastin collagen network, one could speculate that the SNPs on chromosome 9p21.3 might affect the wall integrity either through increased elastin fracture, degradation due to enhanced matrix metalloprotease activity, calcification or enhanced collagen production.

A previous study has reported that central arterial stiffness, as measured by heart-femoral PWV (hfPWV) was more closely associated with CAD, CVD and peripheral arterial disease than peripheral arterial stiffness [26]. hfPWV, carotid-femoral PWV (cfPWV) and afPWV, as used in this study may all be regarded as measures of ‘aortic’ stiffness since in all cases the major part of the path traversed by the pulse wave is the aorta and a recent consensus document confirms that cfPWV is an acceptable estimate of aortic stiffness [27]. However, it should be noted that in older people the ascending aorta lengthens with age [28] as does the common iliac artery, which comprises approximately the distal third of the path [29], and this will add an error to the path length measurements, leading to an overestimation of PWV. Previously, a cut-off point of hfPWV at 11.18 m/s and cfPWV at 12 m/s showed good discrimination in the validation set on the Sphygmocor system and the Colins system, respectively [30]. Deng *et al*. set a cut-off value of cfPWV at ≥ 9 m/s to diagnose arteriosclerosis in patients with essential hypertension [31] and more recently 10 m/s has been proposed as the threshold above which there is an increased risk of cardiovascular events [27]. In the present study, we assessed aortic stiffness in all subjects using the Arterial Compliance Monitor which measures afPWV rather than cfPWV and chose to use our mean value of afPWV (11 m/s). This is close to the values recommended in the consensus document and revealed a clear difference in the association between afPWV and rs1333049 genotypes. We found that the frequency distribution of the rs 1333049 genotypes is significantly associated with afPWV groups (P<0.05). We conclude that, in spite of uncertainties in the precise value of PWV used to dichotomise the data, there is a clear association between polymorphism of rs1333049 and raised arterial stiffness.

In addition to the aforementioned association, we found increased aortic stiffness in subjects homozygous for the C-allele of rs 1333049 on chromosome 9p21.3, after adjusting for age and the number of MetS risk factors. We suggest the SNP rs1333049 might be involved with the progression of raised aortic stiffness in Thais, although the underlying mechanisms by which SNP rs1333049 on chromosome 9p21.3 contributes to this progression remain unclear. Previous studies have suggested that the chromosome 9p21 locus predicts CAD and involves the initiation or facilitation of atherosclerosis [32,33]. Wang *et al*. demonstrated that rs 1333049 polymorphism is an independent determinant of coronary plaque progression in a Chinese Han population [34]. Moreover, it was found that chromosome 9p21 locus is associated with recurrent myocardial infarction or cardiac death following an acute coronary event [35]. Taken together, the genetic variants in this 9p21 locus increase the risk of CAD by promoting atherosclerosis development and plaque instability.

A large noncoding RNA gene named ANRIL(*CDKN2B*-*AS*) is expressed in several cells and tissues, where a number of SNPs located at the 5’ end including rs1333049 have been suggested to be associated with CAD [36]. The ANRIL gene possibly coordinates transcriptional regulation of 2 cyclin-dependent kinase inhibitors (*p16*/*CDKN2A* and *p15*/*CDKN2B*) under both physiological and pathological conditions. A SNP rs1333049 was located in a 190 kb region of the high linkage disequilibrium, upstream both of *p16*/*CDKN2A*, *p15*/*CDKN2B* and *p14*/*ARF* that code for p15 and p16 and p14ARF [36]. These proteins are involved in cell cycle regulation, and also affect the atherosclerotic process by inhibiting the transformation of tumour growth factor-β, a well known tumour suppressor protein [37]. SNP rs1333049 has not only been reported to increase the risk of CAD [38,39] but also of abdominal aortic aneurysms [16,40], these diseases being preceded by altered arterial stiffness. Therefore, our finding of the homozygous CC genotype of SNP rs1333049 in subjects with high aortic stiffness is in good agreement with those observed in CVD patients.

## Conclusions

We have observed that the SNP rs1333049 on chromosome 9p21 locus is associated with increased arterial stiffness in a Thai population. This association is strengthened by the presence of MetS risk. Research on this genetic polymorphism in a large population is required to investigate this association in more detail. Moreover, a mechanistic study is also needed to clarify the effect of the chromosome 9p21 locus on arterial stiffening. Since MetS-associated arterial stiffening increases cardiovascular risk, our findings add to the evidence that management of MetS to prevent advanced cardiovascular complications is clinically important.

## Abbreviations

MetS: Metabolic syndrome; SNP: Single nucleotide polymorphism; IDF: International Diabetes Federation; NCEP-ATPIII: National Cholesterol Education Program Adult Treatment Panel III; afPWV: Aortic-femoral pulse wave velocity; baPWV: Brachial-ankle pulse wave velocity; aaPWV: Aortic-ankle pulse wave velocity; HDL-c: High-density lipoprotein cholesterol; LDL-c: Low-density lipoprotein cholesterol; HOMA-IR: Homeostasis model assessment of insulin resistance; BMI: Body mass index; hsCRP: High sensitivity C-reactive protein.

## Competing interests

The authors declare that they have no competing interests.

## Authors’ contributions

UK and SEG designed the study, analyzed data, reviewed and edited the manuscript. VK performed statistical analysis and edited the manuscript. SP wrote the manuscript and performed experiments. PP, LS, CS, NK and NS performed some experiments. PT, PI and VS participated in research design and were responsible for subjects’ recruitment and the clinical report. All authors read and approved the final manuscript.
